# Pseudoprogression after CAR T-cell therapy mimicking orbital lymphoma relapse

**DOI:** 10.1093/qjmed/hcag037

**Published:** 2026-02-02

**Authors:** Takashi Miyachi, Akihiro Kitadate, Naoto Takahashi

**Affiliations:** Department of Hematology, Nephrology, and Rheumatology, Akita University Graduate School of Medicine, Akita, Japan; Department of Hematology, Nephrology, and Rheumatology, Akita University Graduate School of Medicine, Akita, Japan; Department of Hematology, Nephrology, and Rheumatology, Akita University Graduate School of Medicine, Akita, Japan

A 50-year-old man with relapsed/refractory diffuse large B-cell lymphoma involving the left orbit and maxillary sinus received chimeric antigen receptor (CAR) T-cell therapy with axicabtagene ciloleucel after lymphodepleting chemotherapy. Prior therapies included immunochemotherapy and radiotherapy, achieving a partial response before CAR T-cell infusion. He developed grade 1 cytokine release syndrome on days 5 and 7, treated with tocilizumab and dexamethasone. On day 12, he complained of painless periorbital swelling. Physical examination revealed a soft, non-tender mass around the left eye (Figure 1A). Magnetic resonance imaging showed a heterogeneously contrast-enhanced mass at the site of prior lymphoma involvement, raising concern for disease relapse. However, diffusion-weighted imaging showed no diffusion restriction, with no corresponding signal decrease on the apparent diffusion coefficient map, suggesting relatively low cellularity (Figure 1B and C).

**Figure 1.**
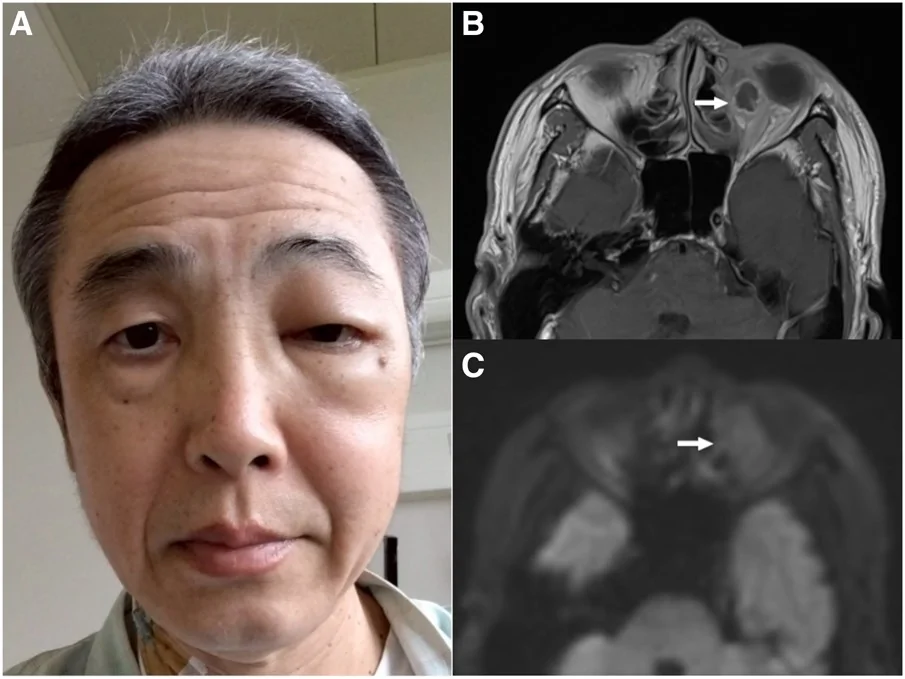
(**A**) Frontal photograph obtained on day 12 after CAR T-cell infusion shows painless left periorbital swelling. Magnetic resonance imaging obtained on the same day shows a heterogeneous ring-enhancing lesion in the left inferior orbit at the site of prior lymphoma involvement (**B**), while corresponding diffusion-weighted magnetic resonance imaging shows no diffusion restriction within the lesion (**C**).

This finding contrasted with the initial lymphoma lesion, which had shown marked diffusion restriction on diffusion-weighted imaging. Given the risk of bleeding and infection, a biopsy was deferred. The lesion resolved spontaneously by day 17. ^18^F-fluorodeoxyglucose positron emission tomography performed on day 30 confirmed complete metabolic remission.

These findings were consistent with pseudoprogression, a phenomenon observed after immunotherapy, characterized by transient tumour enlargement due to infiltration of immune cells. Pseudoprogression occurs in approximately 5–10% of patients receiving immune checkpoint inhibitors.^1^ Although rare, pseudoprogression following CAR T-cell therapy has been reported in lymphoma, with a median onset of 5–7 days (range, 2–21 days).^2^

Because it closely mimics true progression, pseudoprogression poses a significant diagnostic challenge and may lead to unnecessary investigations or premature changes in treatment. Moreover, local immune-mediated inflammation or compressive effects of the enlarged mass can potentially damage adjacent organs, with life-threatening consequences.^3^ Awareness among physicians is needed. Physical examination findings and magnetic resonance imaging, particularly the absence of diffusion restriction despite contrast enhancement, may help distinguish pseudoprogression from true progression.

## Data Availability

Additional clinical information is available upon reasonable request.
